# Influence of Microbial Transglutaminase on Physicochemical and Cross-Linking Characteristics of Individual Caseins

**DOI:** 10.3390/molecules25173992

**Published:** 2020-09-02

**Authors:** Chun-Chi Chen, Liang-Yu Chen, Der-Sheng Chan, Bang-Yuan Chen, Hsien-Wei Tseng, Jung-Feng Hsieh

**Affiliations:** 1 Department of Biological Science and Technology, School of Life Sciences, Longyan University, Longyan 364012, China; kennath1980@gmail.com; 2 Key Laboratory of Preventive Veterinary Medicine and Biotechnology, Longyan University, Longyan 364012, China; 3 Department of Food Science, Fu Jen Catholic University, New Taipei City 242, Taiwan; winniepig10@gmail.com (L.-Y.C.); 125684@mail.fju.edu.tw (B.-Y.C.); 4 Department of Information Technology, Lee-Ming Institute of Technology, New Taipei City 243, Taiwan; dschan@ms58.hinet.net; 5 Department of Mathematics and Information Engineering, Longyan University, Longyan 364012, China; hsienwei.tseng@gmail.com; 6 Ph.D. Program in Nutrition & Food Science, Fu Jen Catholic University, New Taipei City 242, Taiwan

**Keywords:** transglutaminase, κ-casein, α_S_-casein, β-casein, cross-linking

## Abstract

The effects of microbial transglutaminase (MTGase) cross-linking on the physicochemical characteristics of individual caseins were investigated. MTGase was used to modify three major individual caseins, namely, κ-casein (κ-CN), α_S_-casein (α_S_-CN) and β-casein (β-CN). The SDS-PAGE analysis revealed that MTGase-induced cross-linking occurred during the reaction and that some components with high molecular weights (>130 kDa) were formed from the individual proteins κ-CN, α_S_-CN and β-CN. Scanning electron microscopy (SEM) and particle size analysis respectively demonstrated that the κ-CN, α_S_-CN and β-CN particle diameters and protein microstructures were larger and polymerized after MTGase cross-linking. The polymerized κ-CN (~749.9 nm) was smaller than that of β-CN (~7909.3 nm) and α_S_-CN (~7909.3 nm). The enzyme kinetics results showed *K*_M_ values of 3.04 × 10^−6^, 2.37 × 10^−4^ and 8.90 × 10^−3^ M for κ-CN, α_S_-CN and β-CN, respectively, and, furthermore, *k*_cat_ values of 5.17 × 10^−4^, 1.92 × 10^−3^ and 4.76 × 10^−2^ 1/s, for κ-CN, α_S_-CN and β-CN, respectively. Our results revealed that the cross-linking of β-CN catalyzed by MTGase was faster than that of α_S_-CN or κ-CN. Overall, the polymers that formed in the individual caseins in the presence of MTGase presented a higher molecular weight and larger particles.

## 1. Introduction

Milk proteins include caseins and whey proteins and provide essential nutrition for health, development and growth. Caseins are synthesized in the mammary gland and consist of three major milk protein groups, κ-casein (κ-CN), α_S_-casein (α_S_-CN) and β-casein (β-CN). Several whey proteins such as β-lactoglobulin and α-lactalbumin are also synthesized in the mammary gland. However, other whey proteins, including immunoglobulins and bovine serum albumin (BSA), are derived from the blood [1,2]. Many types of dairy products, such as cheese, are made with milk proteins. Traditionally, cheese has been manufactured from milk using enzymes such as chymosin and microbial transglutaminase (MTGase), which are commonly used to improve the properties of cheese products [3,4,5].

MTGase (EC 2.3.2.13) has been widely applied to modify the heat stability and solubility of proteins in food processing [6]. MTGase catalyzes an acyl transfer reaction between a primary amine (acyl acceptor) and the γ-carboxyamide moiety of a protein-bound glutamine residue (acyl donor). When lysine residues act as acyl acceptors, ε-(γ-glutamyl)lysine covalent bonds are formed in proteins, leading to intra- and inter-molecular cross-linking [7]. It has been reported that the polymerization of milk proteins with MTGase results in the formation of a protein film that improves the functional properties of dairy products [8]. Investigations carried out by Agyare and Damodaran [9] revealed that MTGase can modify the heat stability of caseins, as well as their rheological, renneting and gelation properties.

In addition, MTGase is used to improve the properties of products of the cheese manufacturing process [10,11]. Milk proteins are excellent substrates for MTGase, and the cross-linking of milk proteins induced by MTGase provides a totally nontoxic approach to reinforce the structure of protein particles and assemblies. In particular, because of their elastic, random coil structures, caseins are readily cross-linked and lack disulfide bonds in the case of α_S_-CN and β-CN. Monogioudi et al. [12] reported that cross-linked β-CN remained stable under acidic conditions, and its resistance to pepsin digestion exceeded that of non cross-linked β-CN. By contrast, due to the compact globular structures of whey proteins, they tend to cross-link less efficiently [13].

In dairy products, regarding the performance of MTGase, it is vital to understand its reaction with milk proteins in their natural states. Additionally, there is a need to understand the cross-linking of individual casein and whey proteins catalyzed by MTGase. Previous studies have investigated the cross-linking behavior of milk proteins. For example, there are studies on the effect of MTGase on caseins [14,15] and whey proteins [16,17]. However, information on the relative susceptibility of individual caseins to cross-linking is lacking. Thus, our objective in the current study was to investigate the influence of MTGase on the cross-linking and the physicochemical characteristics of individual caseins using SDS-PAGE, particle size, SEM, and enzyme kinetic analyses.

## 2. Results and Discussion

### 2.1. Electrophoretic Profiles of Individual Milk Proteins Cross-Linked by MTGase

**Figure 1:**
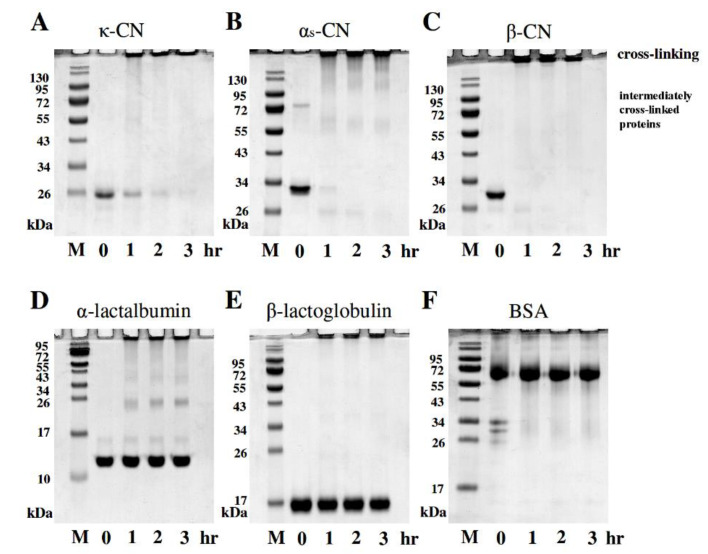
Changes in the SDS-PAGE profiles of milk proteins with 2.0 units/mL microbial transglutaminase (MTGase) at 30 °C for 0, 1, 2, or 3 h. SDS-PAGE gels were loaded using 5 μL milk samples with protein concentrations of 10 μg/well. (**A**) κ-casein (κ-CN) with MTGase; (**B**) α_S_-casein (α_S_-CN) with MTGase; (**C**) β-casein (β-CN) with MTGase; (**D**) α-lactalbumin with MTGase; (**E**) β-lactoglobulin with MTGase; and (**F**) bovine serum albumin (BSA) with MTGase; M = protein marker.

The SDS-PAGE analysis showed the results of the milk proteins (2 mg/mL), including caseins (κ-CN, α_S_-CN and β-CN) and whey proteins (α-lactalbumin, β-lactoglobulin and BSA), after incubation with 2.0 units/mL MTGase for 0, 1, 2, or 3 h at 30 °C. Figure 1A–C present the gels of the casein proteins κ-CN (25 kDa), α_S_-CN (32 kDa) and β-CN (30 kDa) after reaction with 2.0 units/mL MTGase, respectively. Initially, there were no significant changes in the milk protein samples with MTGase in the 0 h incubation period, indicating that cross-linking of κ-CN, α_S_-CN and β-CN did not immediately occur. As shown in Figure 1, intermediately cross-linked individual κ-CN and α_S_-CN were observed following treatment with MTGase for 1 h. The dimer of κ-CN, the dimer of α_S_-CN and the trimer of α_S_-CN were observed in the SDS-PAGE results; however, these protein bands were light and indistinct. Note that we also observed fully cross-linked individual β-CN, for which no dimers or trimers were observed. Moreover, these results revealed little or no cross-linking of α-lactalbumin, β-lactoglobulin, and BSA after reacting with MTGase for 1 h. However, after 3 h of reaction time of κ-CN, α_S_-CN and β-CN with MTGase, the casein protein bands were observed to disappear, with concomitant appearance of new bands of higher molecular weight (>130 kDa, *p* < 0.05) and the accumulation of protein polymers at the stacking and running gel origin. Therefore, the SDS-PAGE analysis results for the casein proteins, as previously mentioned, show that MTGase is an acyltransferase that forms intra- and inter-molecular cross-links by isopeptide bond formation between peptidic lysine and glutamine residues, resulting in cross-linking reactions with milk proteins [7]. Moreover, the MTGase-induced cross-linking reactions with β-CN were faster than that with α_S_-CN or κ-CN because β-CN contains more prolyl residues than other caseins, which gives it a more disordered, flexible and open structure that could facilitate the enzymatic reaction [18]. Furthermore, Figure 1D–F show the SDS-PAGE results of the whey proteins α-lactalbumin (13 kDa), β-lactoglobulin (17 kDa) and BSA (65 kDa) after incubation with 2.0 units/mL MTGase, respectively. The results showed little or no cross-linking of α-lactalbumin, β-lactoglobulin or BSA after 3 h of reaction time with MTGase. Jovanović et al. [19] indicated that whey protein includes the following globular proteins: α-lactalbumin, β-lactoglobulin and BSA. Consequently, it was suggested that α-lactalbumin, β-lactoglobulin and BSA, due to their compact globular structures, tend to cross-link less efficiently and are poor substrates during the reaction with MTGase [13,20].

### 2.2. Cross-Linking Reactions and Enzyme Kinetics and of Individual Caseins by MTGase

**Figure 2:**
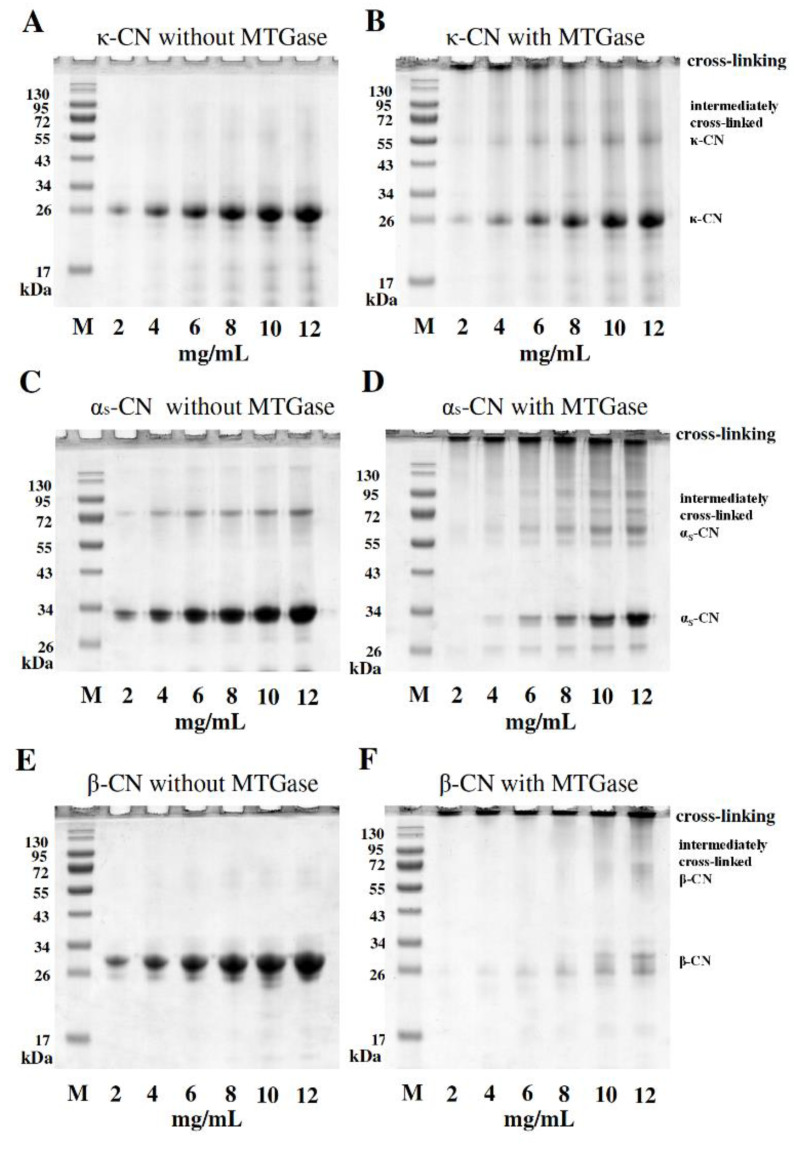
SDS-PAGE profiles from the cross-linking reaction of varying amounts of casein proteins (2–12 mg/mL) without/with 2.0 units/mL MTGase at 30 °C for 1 h. SDS-PAGE gels were loaded using 5 μL individual caseins with protein concentrations of 10, 20, 30, 40, 50, and 60 μg/well, respectively. (**A**) κ-CN without MTGase; (**B**) κ-CN with MTGase; (**C**) α_S_-CN without MTGase; (**D**) α_S_-CN with MTGase; (**E**) β-CN without MTGase; and (**F**) β-CN with MTGase; M: protein marker.

The individual casein proteins κ-CN, α_S_-CN and β-CN (2, 4, 6, 8, 10 and 12 mg/mL) were treated without/with 2.0 units/mL MTGase at 30 °C for 1 h prior to analysis by SDS-PAGE. Table 1 lists the physicochemical features of the individual caseins, including the molecular weight (Da) and glutamine/lysine residues. Note that the glutamine and lysine residues functioned as substrates for MTGase. Figure 2 indicates the SDS-PAGE electrophoretic profiles and protein band intensities of varying amounts of caseins without/with MTGase incubation for κ-CN (Figure 2A,B), α_S_-CN (Figure 2C,D) and β-CN (Figure 2E,F). From these results, there were no components or significant changes observed in the higher molecular weight region (>130 kDa) in the absence of MTGase (Figure 2A,C,E). However, intermediately and fully cross-linked individual κ-CN, α_S_-CN, and β-CN were observed after a 1-h reaction with MTGase. The cross-linking of κ-CN, α_S_-CN and β-CN by MTGase into higher molecular weight proteins (>130 kDa, *p* < 0.05) was observed. Components of higher molecular weights clearly appeared, and were produced by cross-linking of κ-CN, α_S_-CN and β-CN because MTGase catalyzes an acyl transfer reaction between the γ-carboxyamide of a protein-bound glutamine residue and a primary amine [21]. Hence, the result demonstrated that, due to the less ordered conformation and relatively high glutamine and lysine content of casein proteins compared to that of whey proteins, they are excellent substrates for MTGase. Of the casein proteins, β-CN appeared to be the most susceptible to the MTGase-induced reaction. This result also proved that β-CN is a good substrate for MTGase [7].

The relationship between the linear regression Michaelis–Menten model and the SDS-PAGE results of the MTGase-containing milk protein samples was evaluated in this study. The data correspond to SDS-PAGE analysis of the MTGase-containing milk protein samples. The resulting kinetic parameters (*V*_max_, *K*_M_ and *k*_cat_) of MTGase and the substrates (κ-CN, α_S_-CN and β-CN) from Hanes diagrams are shown in Table 2. The *V*_max_ values of 2.73 × 10^−8^, 1.01 × 10^−7^ and 2.52 × 10^−6^ M/sec were found for κ-CN, α_S_-CN and β-CN, respectively. Furthermore, the *K*_M_ values of 3.04 × 10^−6^, 2.37 × 10^−4^ and 8.90 × 10^−3^ M were found for κ-CN, α_S_-CN and β-CN, respectively. *K*_M_ is simply the dissociation constant for the MTGase-casein complex. The greater the value of *K*_M_, the less tightly the casein is bound to the MTGase. We also found *k*_cat_ values of 5.17 × 10^−4^, 1.92 × 10^−3^ and 4.76 × 10^−2^ 1/s, for κ-CN, α_S_-CN and β-CN, respectively. The *k*_cat_ is related to the turnover number of MTGase, which is the number of moles of casein that react to form product per mole of MTGase per second. In our results, we did not observe any precipitate of cross-linked individual caseins catalyzed by MTGase. This is an indication that the single association complex of MTGase and individual caseins could be used as a rapid equilibrium model for our analysis. Nonetheless, it should be noted that linear regression based on Michaelis–Menten kinetics is limited to brief reactions involving a single enzyme and a single substrate of high concentration with rapid equilibrium between the enzyme and substrate. In instances where the product is not soluble, the binding equilibrium between the enzyme and substrate could be disturbed by the accumulation of the reaction product(s) on the active sites. According to these results, the enzyme reaction kinetics of β-CN are faster than those of α_S_-CN and κ-CN in the MTGase-induced cross-linking reaction.

### 2.3. Particle Size Analysis of Individual Caseins Polymerized after MTGase Induction

**Figure 3:**
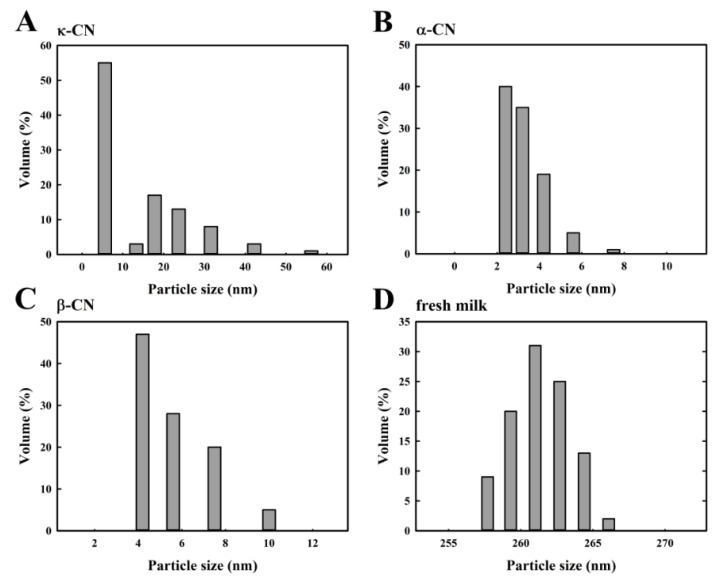
Changes in the particle size distribution of milk proteins without MTGase. (**A**) κ-CN; (**B**) α_S_-CN; (**C**) β-CN; (**D**) fresh milk. The z-average diameter of the colloidal dispersions was measured using a nanoparticle analyzer. Samples were diluted with a buffer (pH 6.8) prior to measurements to avoid multiple scattering effects.

**Figure 4:**
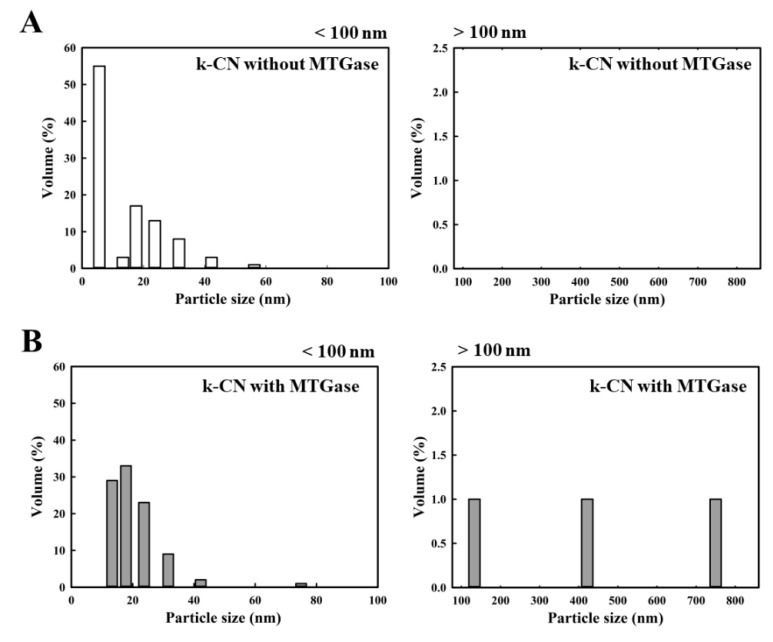
Changes in the particle size distribution of κ-CN (2 mg) without/with 2.0 units/mL MTGase at 30 °C for 3 h. (**A**) κ-CN without MTGase (below 100 nm/above 100 nm); (**B**) κ-CN with MTGase (below 100 nm/above 100 nm). The z-average diameter of the colloidal dispersions was measured using a nanoparticle analyzer. Samples were diluted with a buffer (pH 6.8) prior to measurements to avoid multiple scattering effects.

**Figure 5:**
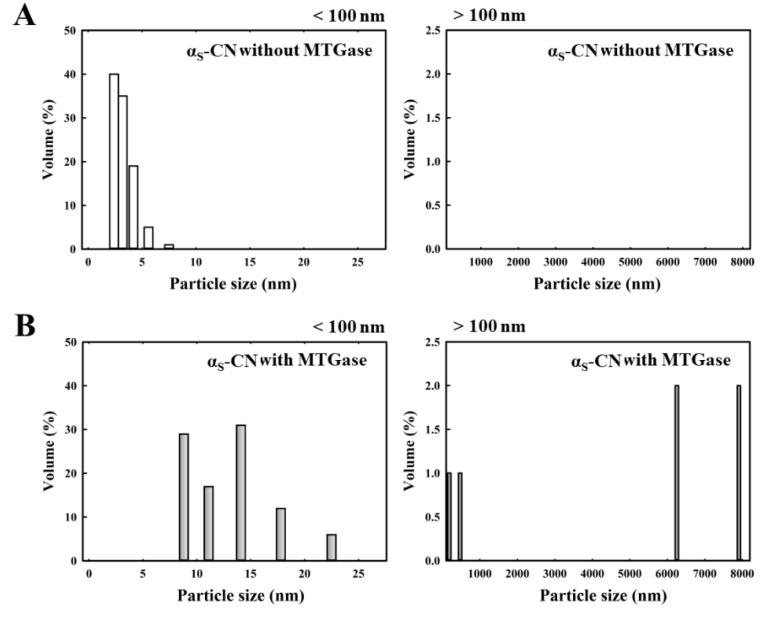
Changes in the particle size distribution of α_S_-CN (2 mg) without/with 2.0 units/mL MTGase at 30 °C for 3 h. (**A**) α_S_-CN without MTGase (below 100 nm/above 100 nm); (**B**) α_S_-CN with MTGase (below 100 nm/above 100 nm). The z-average diameter of the colloidal dispersions was measured using a nanoparticle analyzer. Samples were diluted with a buffer (pH 6.8) prior to measurements to avoid multiple scattering effects.

**Figure 6:**
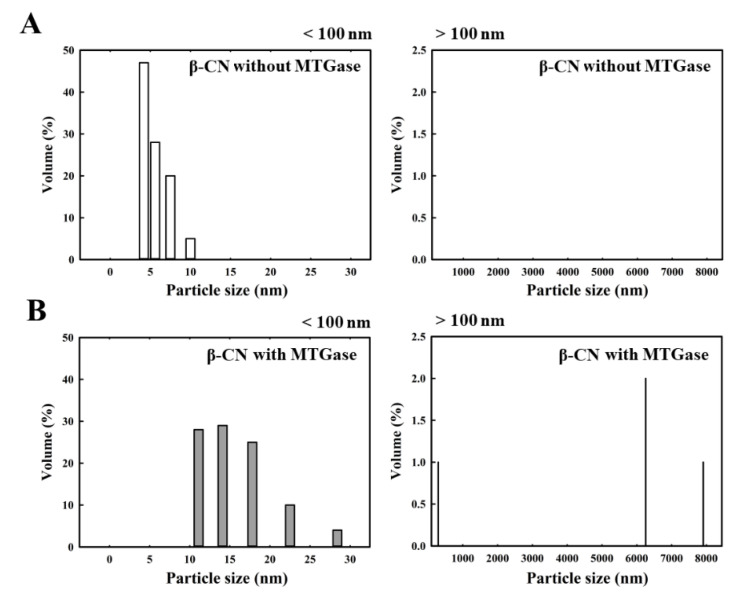
Changes in the particle size distribution of β-CN (2 mg) without/with 2.0 units/mL MTGase at 30 °C for 3 h. (**A**) β-CN without MTGase (below 100 nm/above 100 nm); (**B**) β-CN with MTGase (below 100 nm/above 100 nm). The z-average diameter of the colloidal dispersions was measured using a nanoparticle analyzer. Samples were diluted with a buffer (pH 6.8) prior to measurements to avoid multiple scattering effects.

Figure 3 shows the particle size distribution of the milk proteins, κ-CN (Figure 3A), α_S_-CN (Figure 3B), β-CN (Figure 3C), and fresh milk (Figure 3D) in the absence of MTGase. The particle size distribution of κ-CN, α_S_-CN, β-CN and fresh milk ranges from 5.6 to 56.2 nm, 2.4 to 7.5 nm, 4.2 to 10.0 nm and 257.7 to 266.1 nm, respectively. Because the average sizes depend on the milk composition, the micelle diameters range from less than 100 nm to more than 300 nm in the milk [23]. Additionally, β-CN is mostly present in the interior of the micelle, α_S_-CN is found throughout the micelle structure, and κ-CN is predominantly, if not completely, on the micelle surface [24]. The effect of MTGase on the particle size distribution of κ-CN (Figure 4), α_S_-CN (Figure 5) and β-CN (Figure 6) proteins was measured. The casein proteins κ-CN, α_S_-CN and β-CN without/with 2.0 units/mL MTGase were incubated at 30 °C for 3 h. To clearly analyze the data, the sizes are divided into two regions: below 100 nm and above 100 nm.

Figure 4B, Figure 5B and Figure 6B show the size distribution of κ-CN, α_S_-CN and β-CN proteins with MTGase. The average protein particle diameters (below 100 nm) of κ-CN, α_S_-CN and β-CN ranged from 13.3 to 75.0 nm, 8.8 to 22.5 nm and 11.1 to 28.4 nm, respectively. By contrast, the average protein particle diameters (above 100 nm) of κ-CN, α_S_-CN and β-CN and with MTGase induction ranged from 133.4 to 749.9 nm, 185.5 to 7909.3 nm and 296.5 to 7909.3 nm, respectively. For the sizes above 100 nm, no polymerized κ-CN (Figure 4A), α_S_-CN (Figure 5A) and β-CN (Figure 6A) were found in the absence of MTGase. We noticed that the polymerized κ-CN (~749.9 nm) was smaller than that of β-CN (~7909.3 nm) and α_S_-CN (~7909.3 nm). Moreover, these results indicated that aggregation of the proteins κ-CN, α_S_-CN and β-CN were induced by MTGase. The protein particle diameters of κ-CN, α_S_-CN and β-CN were larger after reaction with MTGase because the enzyme catalyzes an acyl transfer reaction between the ε-amino groups of lysine residues (acyl acceptors) and the γ-carboxyamide groups of peptide-bound glutamine residues (acyl donors), leading to the formation of covalent cross-links in the milk proteins [7,25].

### 2.4. Microstructures of the Individual Caseins Treated with MTGase

**Figure 7:**
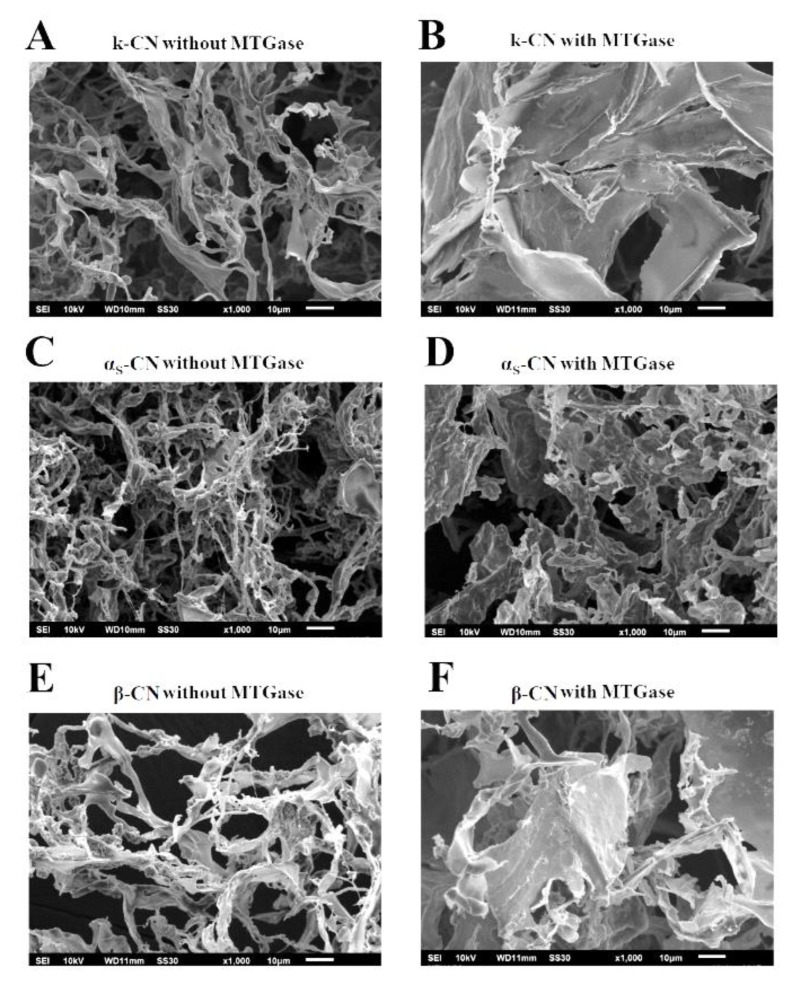
SEM images of casein proteins (2 mg) without/with 2.0 units/mL MTGase at 30 °C for 3 h. (**A**) κ-CN without MTGase; (**B**) κ-CN with MTGase; (**C**) α_S_-CN without MTGase; (**D**) α_S_-CN with MTGase; (**E**) β-CN without MTGase; and (**F**) β-CN with MTGase.

SEM analysis has shown that the structure of foods affects various properties including appearance, functionality and texture. In particular, the microstructure has a major impact on the physical properties and texture of foods. Figure 7 shows that evident changes in the microstructures of casein proteins could be induced after cross-linking by MTGase. The microstructure of κ-CN (Figure 7A,B), α_S_-CN (Figure 7C,D) and β-CN (Figure 7E,F) and without/with cross-linking by MTGase after 3 h incubation was investigated, and SEM micrographs are presented. The results showed that cross-linking of κ-CN, α_S_-CN and β-CN (Figure 7A,C,E) without MTGase generated spongy networks with larger pore sizes and less compact structures. In contrast, the microstructure of the cross-linked protein network is more compact in the MTG-treated samples (Figure 7B,D,F). This finding is in agreement with the results of several authors [26,27,28], who studied the cross-linking of milk proteins with MTGase. These observations indicated that the microstructure of polymers composed of casein proteins could be affected by the MTGase.

## 3. Materials and Methods

### 3.1. Preparation of Milk Protein Samples

The milk protein samples, which included κ-CN, α_S_-CN, β-CN, BSA, β-lactoglobulin and α-lactalbumin, were obtained from Sigma Co. (St. Louis, MO, USA). MTGase was obtained from Ajinomoto Co., Inc. (1.0 units/mg). To investigate the MTGase-induced cross-linking of milk proteins, each milk protein sample and 2.0 units/mL MTGase were added into a 0.05 M phosphate buffer solution (pH 6.8) and dissolved. Each milk protein sample without/with 2.0 units/mL MTGase was incubated in a dry bath incubator for 0, 1, 2, or 3 h at 30 °C. Milk protein samples were heated to 80 °C for 3 min to inactivate the MTGase. Each sample was analyzed in triplicate.

### 3.2. SDS-PAGE Analysis

SDS-PAGE analysis was used to measure the molecular weight of individual caseins with and without MTGase induction according to Hsiao et al. [29]. The SDS-PAGE analysis of milk protein samples used a 5% stacking gel and a 12.5 or 15% separating gel. The milk samples (5 μL) with protein markers (17–130 kDa) were loaded onto the SDS-PAGE gel. Separation was performed in separating gel at 90 V. Following electrophoresis, the gels were stained with Coomassie Brilliant Blue R-250 dye. The gels were imaged and analyzed using the Gel-Pro Analyzer software (version 4.0, Media Cybernetics Inc., Bethesda, MD, USA) and Quantity One 1-D analysis software (version 4.6.3, Bio-Rad Inc., Hercules, CA, USA). Changes in the electrophoretic profiles were used to evaluate the cross-linking of individual caseins induced by MTGase. Each sample was analyzed in triplicate.

### 3.3. Enzyme Kinetics of MTGase Reacting with Individual Caseins

Model and curve fitting: In the MTGase-catalyzed system, the influence of MTGase in the reaction was characterized using the Michaelis–Menten model. The equation used to calculate the reaction rate was as follows:

(1) $$v=\frac{V_{\max}S}{S+K_{m}}$$

where *v* indicates the reaction rate, *S* indicates the substrate concentration, *V_max_* is the maximum initial velocity, and *m* is the Michaelis constant, representing the affinity for enzyme-substrate interaction.

Equation (1) can be rearranged as follows:

(2) $$\frac{S}{v}=\frac{S}{V_{\max}}+\frac{K_{m}}{V_{\max}}$$

To determine the values of the kinetic parameters (*V*_max_, *K_m_*, and *k*_ca_)_,_ we plotted linear regressions results using the Hanes diagrams developed by Menéndez et al. [30]. The MTGase-catalyzed reaction with individual caseins was investigated at various substrate concentrations (from 2 to 12 mg/mL). Using Hanes diagrams, $\frac{S}{v}$ was plotted against *S* to obtain 1/*V*_max_ from the value of the slope and *k_m_*/*V*_max_ from the intercept in order to derive the value of $K_{cat}$ based on the following relation: $K_{cat}=\frac{V_{\max}}{Eo}$, where *Eo* indicates the concentration of MTGase. In the above calculations, *V_max_*, *k_m_*, and $K_{cat}$ are apparent values, due to the cross-linked structure of the individual caseins.

### 3.4. Particle Size Analysis of Polymerized Individual Caseins after MTGase Induction

A particle size analyzer (90 Plus Particle Size Analyzer, Brookhaven Instruments Corporation, Holtsville, NY, USA) was used to determine the particle size range of the milk proteins along with the polydispersity. The particle size was determined by measuring the time-dependent fluctuation of laser light scattered by the nanoparticles undergoing Brownian motion. The MTGase-induced cross-linking of the casein milk protein samples κ-CN, α_S_-CN and β-CN were investigated. Each milk protein sample (2 mg) and 2.0 units/mL MTGase were added into a 0.05 M (pH 6.8) phosphate buffer solution and dissolved. Each sample was analyzed in triplicate.

### 3.5. SEM Analysis

The microstructures of casein proteins (2 mg) κ-CN, α_S_-CN, and β-CN, cross-linked by 2.0 units/mL MTGase after 3 h incubation, were observed with SEM. There were two steps to this process. First, all samples were completely dried and lyophilized overnight. Each milk protein sample without/with MTGase treatment was mounted on a bronze stub and sputter-coated with gold (SPI-module sputter coater, SPI supplies). Second, the samples were observed at an acceleration voltage of 15 kV using a scanning electron microscope (JSM-6510, JEOL Co., Ltd., Tokyo, Japan). Each sample was analyzed in triplicate.

### 3.6. Statistical Analysis

The results are presented as mean ± standard deviation. Duncan’s multiple range tests and one-way ANOVA were used to calculate significant differences between treatments. Three determinations were performed for each treatment, and the level of statistical significance was set at *p* < 0.05. All statistical analysis was performed using SAS software (version 13.2, SAS Institute, Inc., Cary, NC, USA).

## 4. Conclusions

In the present study, the influence of MTGase-induced cross-linking of individual κ-CN, α_S_-CN and β-CN proteins on the polymerization properties was investigated. SDS-PAGE, particle size, SEM and enzyme kinetics analyses of the effects of MTGase on the polymerization of milk proteins were performed. These results showed that MTGase catalyzed the formation of high molecular weight polymers in the κ-CN, α_S_-CN and β-CN fractions and that the polymerization of β-CN occurred faster than that of κ-CN and α_S_-CN. Furthermore, of the casein proteins, β-CN was the most susceptible to the MTGase-induced reaction. This result also proved that β-CN is a good substrate for MTGase.

## Figures and Tables

**Table 1:** Physicochemical features of individual caseins.

Casein Proteins	Molecular Weight (Da)	Glutamine/Lysine Residue
κ-CN	19,037	12/9
α_S_-CN	25,226	25/14
β-CN	24,023	19/11

These data were obtained from Farrell et al. [22].

**Table 2:** Kinetic values of the reaction between 2.0 units/mL MTGase and individual caseins.

Parameter	κ-CN	α_S_-CN	β-CN
*V*_max_ (M/sec)	2.73 × 10^−8^	1.01 × 10^−7^	2.52 × 10^−6^
*K*_M_ (M)	3.04 × 10^−6^	2.37 × 10^−4^	8.90 × 10^−3^
*k*_cat_ (1/s)	5.17 × 10^−4^	1.92 × 10^−3^	4.76 × 10^−2^

Values given are the mean of three measurements.
